# Induction of Broad and Polyfunctional HIV-1-Specific T Cell Responses by the Multiepitopic Protein TMEP-B Vectored by MVA Virus

**DOI:** 10.3390/vaccines7030057

**Published:** 2019-06-29

**Authors:** Beatriz Perdiguero, Cristina Sánchez-Corzo, Carlos Oscar S. Sorzano, Pilar Mediavilla, Lidia Saiz, Mariano Esteban, Carmen Elena Gómez

**Affiliations:** 1 Department of Molecular and Cellular Biology, Centro Nacional de Biotecnología, Consejo Superior de Investigaciones Científicas (CNB-CSIC), Campus de Cantoblanco, 28049 Madrid, Spain; 2 Biocomputing Unit, Centro Nacional de Biotecnología, Consejo Superior de Investigaciones Científicas (CNB-CSIC), Campus de Cantoblanco, 28049 Madrid, Spain

**Keywords:** HIV-1 vaccine, MVA vector, T cell multiepitopes, immunogenicity in mice, T cell responses

## Abstract

A human immunodeficiency virus (HIV)/acquired immune deficiency syndrome (AIDS) vaccine able to induce long-lasting immunity remains a major challenge. We previously designed a T cell multiepitopic immunogen including protective conserved epitopes from HIV-1 Gag, Pol and Nef proteins (TMEP-B), that induced potent HIV-1-specific CD8 T cells when vectored by DNA and combined with the vaccine candidate modified vaccinia virus Ankara (MVA)-B. Here, we described the vectorization of TMEP-B in MVA (MVA-TMEP) and evaluated the T cell immunogenicity profile elicited in mice when administered in homologous (MVA/MVA) or heterologous (DNA/MVA) prime/boost vector regimens or using homologous or heterologous inserts. The heterologous vector regimen was superior to the homologous protocol in inducing T cell responses. DNA-TMEP-primed animals boosted with MVA-TMEP or MVA-B exhibited the highest magnitudes of HIV-1-specific CD8, CD4 and T follicular helper (Tfh) cells, with MVA-TMEP significantly expanding Gag-specific CD8 T cell responses. In the homologous vector regimen, all groups exhibited similar HIV-1-specific CD8 and CD4 T cell responses, but both MVA-B/MVA-B and MVA-TMEP/MVA-TMEP combinations elicited higher Gag-Pol-Nef (GPN)-specific CD8 T cell responses compared to MVA-TMEP/MVA-B. Our results revealed an enhanced induction of HIV-1-specific T cell responses by TMEP-B when vectored in both DNA and MVA, and supported their use in combined prime/boost strategies for HIV-1 prevention and/or therapy.

## 1. Introduction

Since the discovery of human immunodeficiency virus (HIV) more than 30 years ago as the causative agent of acquired immune deficiency syndrome (AIDS), the virus has spread worldwide to pandemic proportions. According to the Joint United Nations Programme on HIV/AIDS (UNAIDS) at the end of 2017 about 36.9 million people globally were living with HIV. Although the increased access to antiretroviral therapy (ART) has markedly reduced both new infections (47% since the peak in 1996) and AIDS-related deaths (51% since the peak in 2004), the development of an effective vaccine remains to be a priority in the field (http://www.unaids.org).

Natural immunity and vaccine immune correlates of protection against HIV are not well defined yet, but there is an agreement that an ideal candidate should be able to mobilize both cellular and humoral immune responses. The challenge for such vaccine development is that viral antigens, delivery systems, adjuvants, cytokines and chemokines that promote robust antibody responses are generally not the same as those that promote robust T cell responses, hindering the generation of a single vaccine candidate that elicits both types of protective responses at the same time. Moreover, the large sequence diversity of circulating HIV-1 strains and the rapid seeding of intracellular DNA reservoirs are key elements that further increase the complexity for immunogen design. The current ongoing preclinical and clinical trials combining different antibody- and T cell-inducing vaccine candidates using homologous or heterologous prime-boost strategies is the most realistic option to overcome the above challenge and confer enhanced protection against HIV infection [1,2].

All viral proteomes contain conserved regions that are mainly found in those proteins where functional constraints limit wide sequence variability. In fact, several groups have focused immune recognition towards such conserved regions to improve vaccine efficacy [3,4,5,6,7,8]. Different strategies have been developed to define the best viral antigen target epitopes. These strategies include consensus, mosaic and conserved sequence approaches as well as networked, functional and critical function epitope approaches [9].

We have previously reported the design of a T cell multiepitopic-based immunogen, termed TMEP-B, comprising eight HIV-1 fragments including multiple beneficial CD4 and CD8 T cell epitopes, mostly derived from conserved regions in Gag, Pol and Nef proteins, that are restricted by a wide range of Human Leukocyte Antigen (HLA) class I and II molecules and that have been functionally associated with low viral load and HIV-1 control [10]. TMEP-B expressed from a DNA vector (DNA-TMEP) induced potent HIV-1-specific CD8 T cell responses in mice when administered in combination with modified vaccinia virus Ankara (MVA)-B, an HIV-1 vaccine that simultaneously expresses the full-length HIV-1 Gag-Pol-Nef (GPN) fusion protein and the monomeric gp120 of clade B [11]. In phase I clinical trials, both prophylactic and therapeutic MVA-B vaccine administered as three homologous doses showed moderated immunogenicity profiles against HIV-1 antigens, with most of the T cell responses Env-driven [12,13,14]. In the MVA-B therapeutic clinical trial there was some reduction in viral load after ART-treatment withdrawal, but not sufficient to control virus replication [15,16]. These findings suggested that generation of a more potent T cell immune response against HIV-1 antigens by vaccination was needed.

In an effort to further improve the HIV-1 specific T cell responses to conserved HIV-1 regions in a prime/boost protocol, here we describe the vectorization of TMEP-B in MVA vector to generate the MVA-TMEP recombinant virus, and evaluate the T cell immunogenicity profile elicited in mice when administered both in homologous (MVA/MVA) or heterologous (DNA/MVA) prime/boost protocols or using homologous or heterologous inserts. Our findings show that prime/boost with DNA-TMEP/MVA-TMEP is an effective protocol to trigger broad and polyfunctional T cell responses (CD8, CD4 and T follicular helper (Tfh) cells) against HIV-1 Gag, Pol and Nef proteins, immune parameters which might be relevant to enhance control of HIV-1 infection.

## 2. Materials and Methods

### 2.1. Cells and Viruses

Human HeLa cells (epithelial cervix adenocarcinoma cells; ATCC CCL-2), primary chicken embryo fibroblast (CEF) cells (obtained from pathogen-free 11-day-old eggs; MSD, Salamanca, Spain) and DF-1 cells (a spontaneously transformed CEF cell line; ATCC CRL-12203) were grown in Dulbecco’s modified Eagle’s medium (DMEM) supplemented with 2 mM L-glutamine (Merck, Kenilworth, NJ, USA), 100 U/mL penicillin/100 µg/mL streptomycin (Sigma-Aldrich, St. Louis, MO, USA), 0.1 mM non-essential amino acids (Sigma-Aldrich), 0.5 μg/mL amphotericin B (Fungizone; Gibco-Life Technologies, Waltham, MA, USA) and 10% newborn calf serum (NCS; Sigma-Aldrich) for HeLa cells or 10% heat-inactivated fetal calf serum (FCS; Sigma-Aldrich) for CEF and DF-1 cells. The different cell lines were maintained at 37 °C in a humidified air, 5% CO_2_ atmosphere.

The viruses used in this work included: the attenuated wild-type modified vaccinia virus Ankara (MVA-WT) obtained from the Ankara strain after 586 serial passages in CEF cells (provided by G. Sutter), the MVA-B recombinant virus that simultaneously expresses the full-length HIV-1 Gag–Pol–Nef (GPN) fusion protein as an intracellular product and the monomeric gp120 as a cell-released product of clade B from the viral thymidine kinase (TK) locus (*J2R* gene) [11] and the novel MVA-TMEP-B (shortly MVA-TMEP) that expresses TMEP-B protein [10] from the TK locus. All virus infections were performed with DMEM-2% FCS or NCS.

### 2.2. DNA Vectors

Plasmids pcDNA-TMEP-B (shortly DNA-TMEP), used as priming agent for in vivo assays, and pCyA-20-TMEP-B (shortly pCyA-TMEP), used as a plasmid transfer vector for the generation of the recombinant virus MVA-TMEP in which the TMEP-B gene was inserted into the viral TK locus of the parental MVA-WT virus, have been previously described [10].

### 2.3. Construction of MVA-TMEP Recombinant Virus

For the generation of MVA-TMEP recombinant virus, 3 × 10^6^ of primary CEF cells were infected with MVA-WT at a multiplicity of infection (MOI) of 0.05 pfu/cell and transfected 1 h later with 8 µg of pCyA-TMEP using Lipofectamine-2000 (Invitrogen, Carlsbad, CA, USA) and following the manufacturer’s recommendations. At 72 h post-infection (h.p.i.), cells were harvested, lysed by freeze-thaw cycling, briefly sonicated and then used for the screening of MVA recombinant viruses. MVA-based viruses transiently co-expressing the β-galactosidase (β-Gal) marker gene (*lacZ* gene) and containing the TMEP-B gene were isolated after three sequential plaque purification steps in DF-1 cell monolayers that were stained with 5-bromo-4-chloro-3-indolyl β-D-galactopyranoside (X-Gal; 1.2 mg/mL). After the recombinant viruses expressing β-Gal and TMEP-B insert have been isolated, further amplification of this recombinant virus leads to the self-deletion of the β-Gal marker gene by homologous recombination between the TK left arm and the short TK left arm repeat that are flanking the marker gene. Hence, in the subsequent three isolation steps, MVA recombinant viruses having deleted the β-Gal marker gene and containing TMEP-B gene were selected by plaque purification screening for non-stained viral plaques in DF-1 cells in the presence of X-Gal. The resulting MVA-TMEP recombinant virus was grown in CEF cells and the viral crude preparations obtained were used for the amplification of the viruses in large cultures of CEF cells, followed by virus purification through two 36% (w/v) sucrose cushions. The virus titers were determined at least three times by immunostaining plaque assay in monolayers of DF-1 cells, as previously reported [17]. The viral stocks were free of fungi, bacteria and mycoplasma contamination.

### 2.4. PCR Analysis of MVA-TMEP Recombinant Virus

To determine the identity and purity of MVA-TMEP viral preparation, DNA was extracted from DF-1 cells infected with MVA-WT or MVA-TMEP at 5 pfu/cell for 24 h. Cell membranes were disrupted by treatment with proteinase K (0.2 mg/mL proteinase K in 50 mM Tris-HCl pH 8, 100 mM NaCl, 1% sodium dodecyl sulfate (SDS), 100 mM ethylenediamine tetraacetic acid (EDTA) pH 8; 1 h at 55 °C), followed by incubation with RNase A (80 µg/mL). DNA was precipitated with 2-propanol. Primers TK-R: 5′-CTGCCGTATCAAGGACA-3′ and TK-L: 5′-TGATTAGTTTGATGCGATTC-3′ spanning TK flanking regions were used for the analysis of the TK locus by PCR. The amplification reactions were performed with Phusion High-Fidelity DNA polymerase (BioLabs, Ipswich, MA, USA), according to the manufacturer’s instructions.

### 2.5. Analysis of Virus Growth

To analyze the viral growth of MVA-TMEP virus, primary CEF cells grown in 12-well plates were infected with MVA-WT or MVA-TMEP at 0.01 pfu/cell. After virus adsorption for 60 min at 37 °C, the inoculum was removed and the cells were incubated with DMEM-2% FCS in a 5% CO_2_ atmosphere at 37 °C. At different times post-infection (0, 24, 48 and 72 h), the infected cells were collected, centrifuged at 3000 rpm for 5 min, supernatant removed and 0.1 mL of complete DMEM added to the cellular pellet. Cell lysates were freeze-thawed three times and briefly sonicated and virus titers were determined by immunostaining plaque assay in monolayers of DF-1 cells using rabbit polyclonal anti-vaccinia virus (VACV) strain Western Reserve (WR) (1:1000; CNB), followed by goat anti-rabbit-horseradish peroxidase (HRP) (1:1000; Sigma-Aldrich).

### 2.6. Time-Course Expression of TMEP-B Protein by Western-Blot Analysis

To evaluate the correct expression of TMEP-B protein from MVA-TMEP virus, DF-1 cells grown in 24-well plates were infected with MVA-TMEP at 5 pfu/cell. At different times post-infection (0, 4, 8 and 24 h), infected cells were harvested and centrifuged at 3000 rpm for 5 min. The supernatant was collected and 10% trichloroacetic acid (TCA) was added for 1 h on ice to precipitate the proteins and then centrifuged at 3000 rpm for 5 min. The supernatant was discarded and the pellet was washed with acetone, centrifuged at 3000 rpm for 5 min and resuspended in Laemmli buffer with 2-mercaptoethanol. On the other hand, cellular pellets were lysed in cold buffer (50 mM Tris-HCl pH 8, 0.5 M NaCl, 10% NP-40, 1% SDS) on ice for 30 min and centrifuged at 13,000 rpm for 20 min at 4 °C. The supernatant was transferred to a new tube and Laemmli buffer 2X with 2-mercaptoethanol was added. Both supernatant and pellet samples were fractionated by 8% SDS-polyacrylamide gel electrophoresis (PAGE) and analyzed by Western-blot using the rabbit polyclonal anti-p24 Gag serum (1:1000; ARP 432, NIBSC, Centralised Facility for AIDS reagent, Potters Bar, UK), followed by goat anti-rabbit-HRP (1:5000; Sigma-Aldrich) to evaluate the expression of TMEP-B protein. The immunocomplexes were detected by enhanced chemiluminescence (ECL; GE Healthcare, Chicago, IL, USA).

### 2.7. Fractionation of TMEP-B Protein into Different Virion Compartments

Localization of TMEP-B protein in MVA-TMEP virions was determined by sequential detergent treatment as previously reported [18]. Briefly, sucrose cushion-purified virions were resuspended in 0.1 mL of Tris-buffer-NP-40 (50 mM Tris-HCl pH 8.5, 10 mM MgCl_2_, 1% non-ionic detergent NP-40) by sonication. This and subsequent steps were carried out at 37 °C for 30 min. The soluble lipid envelopes (fraction E1) were removed by centrifugation, and the remaining pellet was resuspended in 0.1 mL of Tris-buffer-NP-40 plus 50 mM dithiothreitol (DTT). The soluble protein matrix-like membranes (fraction E2) were harvested after centrifugation, and the pellet was resuspended in 0.1 mL of the previous buffer plus 0.1% SDS and 0.5% deoxycholic acid (DOC). The soluble core proteins (fraction E3) were removed by centrifugation, and the pellet containing the remaining cores (C fraction) was resuspended in 0.1 mL of milliQ H_2_O. The presence of TMEP-B protein in the different fractions (E1, E2, E3 and C) was analyzed by Western-blot using rabbit polyclonal anti-p24 Gag serum.

### 2.8. Detection of TMEP-B Expression by Flow Cytometry

The expression of TMEP-B protein from MVA-TMEP recombinant virus was also determined by flow cytometry using a specific antibody against the FLAG tag located at the C-terminus of the TMEP-B sequence. First, 10^6^ HeLa cells were infected at 5 pfu/cell with MVA-WT or MVA-TMEP viruses. At different times post-infection (0, 4, 8 and 24 h), the cells were washed with phosphate buffered saline (PBS) (Ca^−^/Mg^−^), dissociated with PBS 1X-2 mM EDTA, washed with flow cytometry staining (FACS) buffer (PBS 1X-2 mM EDTA) and centrifuged for 5 min at 1500 rpm. Next, cells were stained with the live/dead fixable red dye (1:200; Invitrogen) at 4 °C in the dark for 30 min, washed twice with FACS buffer and fixed/permeabilized with BD Cytofix/Cytoperm (BD Biosciences, San Jose, CA, USA) at 4 °C for 20 min. Cells were then centrifuged for 5 min at 1500 rpm and left overnight in FACS buffer at 4 °C in the dark. The next day, cells were centrifuged, blocked with PBS 1X-3% bovine serum albumin (BSA) at 4 °C for 30 min and incubated with 5 µg/mL of the monoclonal antibody anti-FLAG M2 (Sigma-Aldrich) in PermWash buffer 1X (PW 1X; BD Biosciences) at 4 °C in the dark for 30 min. The cells were then washed twice with PW 1X and secondary anti-mouse IgG (H+L)-PE antibody (1:100; Beckman Coulter, Brea, CA, USA) in PW 1X was added to the cells. After 30 min of incubation at 4 °C in the dark, the cells were washed twice with PW 1X, resuspended in FACS buffer and acquired in a FC500 1 Laser flow cytometer (Beckman Coulter). Data analyses were carried out using FlowJo software (Version 10.4.2; Tree Star, Ashland, OR, USA). Percentages of FL2^+^ cells on the “live cells” gate and geometric Mean Fluorescence Intensity (gMFI) values were used to analyze the results.

### 2.9. Peptides

The HIV-1 clade B consensus peptide pools used in this work include: Env-1 (63 peptides), Env-2 (61 peptides), Gag-1 (55 peptides), Gag-2 (50 peptides), GPN-1 (53 peptides), GPN-2 (57 peptides), GPN-3 (56 peptides) and GPN-4 (55 peptides). They were provided by the National Institutes of Health (NIH) AIDS Research and Reference Reagent Program (Germantown MD, USA) and covered the HIV-1 clade B Env, Gag, Pol and Nef proteins included in the HIV-1 antigens expressed from MVA-B as consecutive 15-mers overlapping by 11 amino acids. The peptide pools that span the different regions included in TMEP-B protein have been previously described [10]. To analyze the HIV-1-specific cellular immune responses, we combined the above peptide pools as follows: Env pool (Env-1 + Env-2), Gag pool (Gag-1 + Gag-2) and GPN pool (GPN-1 + GPN-2 + GPN-3 + GPN-4). FLAG peptide (sequence: DYKDDDDK; Sigma-Aldrich) was used to detect FLAG-specific T cell responses and VACV E3_140-148_ peptide (sequence: VGPSNSPTF; CNB-CSIC Proteomics Service, Madrid, Spain), previously described as an immunodominant epitope in BALB/c mice [19], was used to determine VACV-specific CD8 T cell responses.

### 2.10. Ethics Statement

The Ethical Committee of Animal Experimentation (CEEA) of Centro Nacional de Biotecnología (CNB-CSIC, Madrid, Spain) approved the animal experimental protocols, according to International EU Guidelines 2010/63/UE on protection of animals used for experimentation and other scientific purposes, Spanish National Law 32/2007 on animal welfare, exploitation, transport and sacrifice and Spanish National Royal Decree RD 53/2013 (permit number: PROEX 014/15).

### 2.11. Mouse Immunization Schedule

Female BALB/c mice (6–8 weeks old; n = 4/group) purchased from ENVIGO were immunized with 1 × 10^7^ pfu of MVA-WT, MVA-TMEP or MVA-B or with 50 μg of DNA-TMEP by bilateral intramuscular (i.m.) route. Four weeks later, the animals were boosted with the same dose of MVA constructions and using the same route of administration. At 10 days post-boost, the mice were euthanized and their spleens were processed for Intracellular Cytokine Staining (ICS) assay to measure the cellular immune response against HIV-1, FLAG or VACV antigens.

### 2.12. Analysis of the Cellular Immune Responses by ICS Assay

#### 2.12.1. Analysis of CD4 and CD8 T Cell Responses

To analyze the magnitude and phenotype of the HIV-1-, FLAG- or VACV-specific T cell immune responses, 2 × 10^6^ splenocytes (erythrocyte-depleted) seeded on 96-well plates were stimulated for 6 h in complete Roswell Park Memorial Institute (RPMI) 1640 medium (100 units/mL of penicillin/100 μg/mL of streptomycin, 2 mM L-glutamine, 10 mM Hepes and 0.01 mM β-mercaptoethanol) with 10% FCS, anti-CD107a-FITC (BD Biosciences), 1 µL/mL Golgiplug (BD Biosciences), monensin 1X (Invitrogen) and 5 µg/mL of the different HIV-1 clade B consensus peptide pools or 5 µg/mL of FLAG peptide or 10 µg/mL of VACV E3 peptide. After stimulation, splenocytes from immunized mice were stained for surface markers, fixed/permeabilized (Cytofix/Cytoperm kit; BD Biosciences) and stained intracellularly with the following fluorochrome-conjugated antibodies: IL-2-APC, IFN-γ-PeCy7 and TNF-α-PE for functional analyses and CD3-PECF594, CD4-APCCy7, CD8-V500, CD127-PerCPCy5.5 and CD62L-Alexa700 for phenotypic analyses (all from BD Biosciences). The dead cells were excluded using the violet LIVE/DEAD stain kit (Invitrogen).

#### 2.12.2. Analysis of Tfh Cell Responses

To determine the magnitude and phenotype of the HIV-1- and FLAG-specific Tfh cell immune responses, 2 × 10^6^ splenocytes (erythrocyte-depleted) seeded on 96-well plates were stimulated for 6 h in complete RPMI 1640 medium with 10% FCS, anti-CD154 (CD40L)-PE (BD Biosciences), 1 µL/mL Golgiplug (BD Biosciences), monensin 1X (Invitrogen) and 5 µg/mL of the different HIV-1 clade B consensus peptide pools or 5 µg/mL of FLAG peptide. After stimulation, the splenocytes from immunized mice were stained for surface markers, fixed/permeabilized (Cytofix/Cytoperm kit; BD Biosciences) and stained intracellularly with the following fluorochrome-conjugated antibodies: IL-4-Alexa488, IFN-γ-PeCy7 and IL-21-APC for functional analyses and CD4-Alexa700, CD8-V500, CXCR5-PECF594 and PD1 (CD279)-APCefluor780 for phenotypic analyses (all from BD Biosciences). The dead cells were excluded using the violet LIVE/DEAD stain kit (Invitrogen).

Cells were acquired in a GALLIOS flow cytometer (Beckman Coulter) and data analyses were carried out using FlowJo software (Version 10.4.2; Tree Star). Lymphocyte-gated events ranged between 10^5^ and 5 × 10^5^. After gating, boolean combinations of single functional gates were generated to quantify the frequency of each response based on all the possible combinations of cytokine expression or differentiation markers. Background responses in the unstimulated controls (RPMI) were subtracted from those obtained in stimulated samples for each specific functional combination.

### 2.13. Data Analysis and Statistics

For the statistical analysis of ICS data, an approach that adjusts the values for the non-stimulated controls (RPMI) and calculates the confidence intervals and *p* values was used [20]. Only antigen responses significantly higher than the corresponding RPMI values were represented. The distribution of the polyfunctional responses was analyzed and represented using SPICE software (version 5.1, downloaded from http://exon.niaid.nih.gov [21]). The different distributions were compared using a Student’s *t*-test and a partial permutation test as previously described [21]. All of the values represented are background-subtracted.

## 3. Results

### 3.1. In Vitro Characterization of MVA-TMEP Recombinant Virus

#### 3.1.1. Purity and Virus Growth

**Figure 1:**
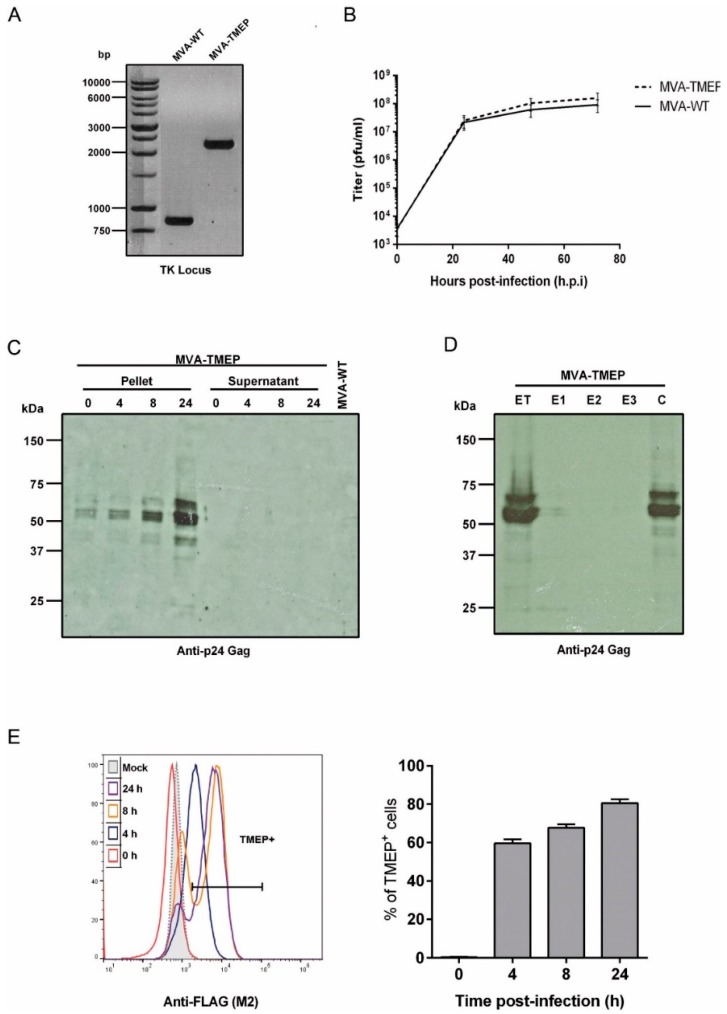
In vitro characterization of modified vaccinia virus Ankara (MVA)-TMEP recombinant virus. (**A**) Confirmation of TMEP-B insertion by PCR analysis. DNA was extracted from DF-1 cells infected at 5 pfu/cell with MVA-wild-type (WT) or MVA-TMEP viruses. Primers thymidine kinase (TK)-R and TK-L spanning TK flanking regions were used for the analysis of the TK locus by PCR. In parental MVA-WT, a product of 853 bp corresponding to parental TK locus is obtained while in MVA-TMEP a unique product of 2292 bp is observed. (**B**) Analysis of virus growth. Monolayers of chicken embryo fibroblast (CEF) cells (permissive cell line) were infected at 0.01 pfu/cell with MVA-WT or MVA-TMEP viruses. At different times post-infection (0, 24, 48 and 72 h), cells were harvested and viral titers were determined by immunostaining plaque assay in DF-1 cells. Data points represent titers as mean ± SD of n = 2 experiments. (**C**) Time-course expression of TMEP-B protein by Western-blot analysis. DF-1 cells were infected at 5 pfu/cell with MVA-WT or MVA-TMEP viruses. At different times post-infection (0, 4, 8 and 24 h), cells were harvested and cellular pellets and supernatants were processed as described in Materials and Methods, fractionated by 8% SDS-PAGE and the expression of TMEP-B protein was analyzed by Western-blot using the rabbit polyclonal anti-p24 Gag serum. (**D**) Fractionation of viral proteins and localization of TMEP-B protein within purified MVA-TMEP virions. MVA-TMEP sucrose-purified virions were sequentially disrupted by detergent treatment and the different fractions were obtained as described under Materials and Methods. The unfractionated lysate virions (total extract, ET) and the different collected fractions (E1, E2, E3 and C) were analyzed by Western-blot using anti-p24 Gag antibody. (**E**) Analysis of TMEP-B expression by flow cytometry. HeLa cells infected at 5 pfu/cell with MVA-WT or MVA-TMEP viruses for 0, 4, 8 and 24 h were processed for flow cytometry as described under Materials and Methods using 5 µg/mL of monoclonal antibody anti-FLAG. Geometric Mean Fluorescence Intensity (gMFI) values (left panel) and percentages of FL2^+^ cells (right panel) on the “live cells” gate are represented.

The correct insertion of TMEP-B construct into the TK locus of MVA genome as well as the purity of the MVA-TMEP viral preparation were analyzed by PCR using primers annealing in the flanking regions of TK locus. As observed in Figure 1A, the TMEP-B gene was successfully inserted into the viral TK locus since the expected 2292 bp product was observed and no parental contamination (853 bp product) was present in MVA-TMEP viral stock. These results were also confirmed by DNA sequence analysis.

Next, a growth curve analysis was performed in primary CEF cells (permissive cell line) to determine whether the insertion of TMEP-B gene affected the viral growth of MVA-TMEP in cell culture. As shown in Figure 1B, the growth kinetics of parental MVA-WT and MVA-TMEP recombinant virus were similar, indicating that the insertion of TMEP-B protein into the MVA-WT genome did not impair its growth properties.

#### 3.1.2. Time-Course Expression of TMEP-B Protein by Western-Blot Analysis

The expression kinetics of TMEP-B protein was analyzed by Western-blot in cellular pellets and supernatants from DF-1 cells infected with MVA-WT or MVA-TMEP viruses for 0, 4, 8 and 24 h. As shown in Figure 1C, the expected 68 kDa product was detected over time. Other protein bands of lower size might represent processed products of TMEP. However, despite the insertion of the tPA-22P/A signal peptide in the TMEP-B construction for protein secretion, we were unable to detect it in the supernatant of infected cells at any time-point analyzed (Figure 1C).

#### 3.1.3. Incorporation of HIV-1 Antigens in Different Compartments within MVA Virions

Since MVA viral preparations used for vaccination consist mainly of intracellular mature virus (IMVs), we next determined whether virus particles purified from MVA-TMEP-infected cells incorporated TMEP-B protein. To do this, we determined the presence of TMEP-B protein after sequential detergent treatment of purified MVA-TMEP preparations. As shown in Figure 1D, TMEP-B protein was only found in the core fraction (C), highlighting the nature of TMEP-B protein. Again, two main protein bands were observed in the gels, indicating that during virus assembly in the cytoplasm those proteins bind tightly to virion core proteins.

#### 3.1.4. Detection of TMEP-B Expression by Flow Cytometry

The expression kinetics of TMEP-B protein from MVA-TMEP virus was also determined in permeabilized cells by flow cytometry using a specific antibody against the FLAG tag incorporated at the C-terminus of the TMEP-B sequence. As observed in Figure 1E, in non-permissive HeLa cells infected with MVA-TMEP virus at 5 pfu/cell, the percentages of TMEP^+^ cells increased with time (0, 4, 8 and 24 h.p.i.), indicating efficient expression of TMEP protein in the course of virus infection.

### 3.2. Immune Response Induced by MVA-TMEP Recombinant Virus in Mice

Next, we examined the immunogenicity elicited in mice by the TMEP-B antigen expressed from DNA (DNA-TMEP) or MVA (MVA-TMEP) following homologous or heterologous prime/boost protocols in combination with the MVA-B vaccine that simultaneously expresses clade B Gag-Pol-Nef (GPN) fusion protein and monomeric gp120. Thus, BALB/c mice (n = 4/group) were immunized as described in Materials and Methods and T cell (CD4, CD8 and Tfh) immune responses were analyzed by ICS assay at 10 days post-boost. The different immunization groups were: DNA-TMEP/MVA-B, DNA-TMEP/MVA-TMEP, MVA-TMEP/MVA-B, MVA-B/MVA-B and MVA-TMEP/MVA-TMEP. Mice primed and boosted with MVA-WT were used as controls. For the detection of the HIV-1-specific T cell responses, splenocytes from immunized mice were stimulated ex vivo for 6 h with a panel of 450 peptides from HIV-1 clade B consensus, covering the full-length sequences of HIV-1 Env, Gag, Pol and Nef proteins included in the MVA-B recombinant vector. The peptides were grouped in three pools: Env pool (Env-1 + Env-2), Gag pool (Gag-1 + Gag-2) and GPN pool (GPN-1 + GPN-2 + GPN-3 + GPN-4). VACV- and FLAG-specific responses were analyzed using VACV E3 or FLAG peptides, respectively.

#### 3.2.1. HIV-1 Gag- and GPN-Specific CD4 and CD8 T Cell Immune Responses Elicited in Mice

Since cellular immune response displays a key role in HIV-1 control, we first evaluated the HIV-1 Gag- and GPN-specific CD4 and CD8 T cell responses elicited in mice by the different immunization protocols. Splenocytes from immunized mice were non-stimulated (RPMI) or stimulated ex vivo for 6 h with the different HIV-1 clade B consensus peptide pools. After stimulation, cells were stained with specific antibodies to identify T cell lineage (CD3, CD4 and CD8) and responding cells based on the secretion of effector cytokines (IFN-γ, IL-2 and TNF) and expression of CD107a as an indirect marker of cytotoxicity. The percentages of CD4 or CD8 T cells that produced IFN-γ and/or IL-2 and/or TNF and/or expressed CD107a established the overall CD4^+^ or CD8^+^ T cell responses.

**Figure 2:**
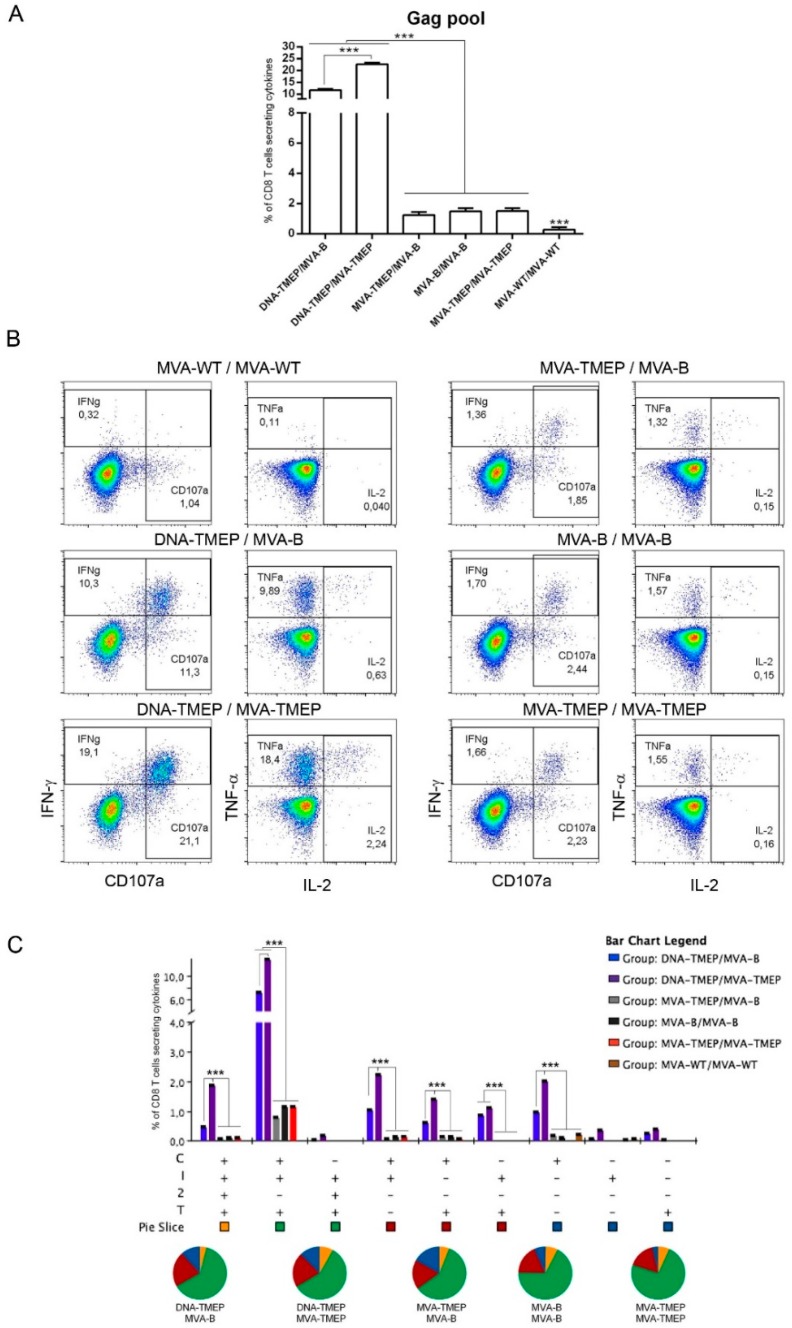
Polyfunctional Gag-specific CD8 T cell responses induced in spleen after prime/boost immunization of mice with different DNA and MVA vectors expressing TMEP-B protein. (**A**) Magnitude of the Gag-specific CD8 T cell responses determined at 10 days post-boost by intracellular cytokine staining (ICS) assay after the stimulation of splenocytes from immunized mice with the human immunodeficiency virus (HIV)-1 clade B consensus Gag pool. The total value in each group represents the sum of the percentages of CD8^+^ T cells secreting IFN-γ and/or IL-2 and/or TNF and/or expressing CD107a against the HIV-1 Gag pool. Data are background-subtracted. (**B**) Flow cytometry profiles of the vaccine-induced CD8 T cell response against the Gag pool in the different immunization groups. (**C**) Polyfunctional profile of the Gag-specific CD8 T cell response. The percentages of the functionally distinct cell populations within the total CD8 T cells are represented on the *y* axis, while positive combinations of the responses are indicated on the *x* axis. Responses are grouped and color-coded based on the number of functions. C: CD107a; I: IFN-γ; 2: IL-2; T: TNF. Data from one experiment representative of two performed are shown and 95% Confidence Interval (CI) is represented. ***, *p* < 0.001.

Regarding Gag-specific CD8 T cell responses (Figure 2), animals immunized with the heterologous DNA/MVA combinations elicited significantly higher Gag-specific CD8 T cells than those induced in animals immunized with the homologous MVA/MVA combinations (*p* < 0.001). Moreover, the group of mice immunized with the homologous insert regimen (DNA-TMEP/MVA-TMEP) elicited significantly higher Gag-specific responses than the group receiving the heterologous insert regimen (DNA-TMEP/MVA-B) (*p* < 0.001), indicating a beneficial effect of the use of identical TMEP-B immunogen both in prime and boost compared to the combination of heterologous TMEP-B and GPN antigens on the outcome of the Gag-specific response. No statistical differences were observed in the responses induced by the groups immunized with the homologous MVA/MVA combinations, independently of the insert regimen they expressed (Figure 2A). Representative flow cytometry profiles of vaccine-induced CD8 T cell responses against the Gag pool in the different immunization groups are shown in Figure 2B. The quality of a specific T cell response can be determined by the profile of cytotoxic potential and cytokine production. Thus, the polyfunctional profile of the Gag-specific CD8 T cell responses was determined based on the analysis of surface mobilization of CD107a on activated T cells as an indirect marker of cytotoxicity and IFN-γ, IL-2 and TNF secretion. Nine positive Gag-specific CD8 T cell populations were induced after immunization with the different prime/boost protocols (Figure 2C). In all groups, Gag-specific responses were highly polyfunctional, with more than 70% of responding CD8^+^ T cells exhibiting three or four functions. CD107a + IFN-γ + TNF-secreting cells was the most representative CD8 T cell population induced (Figure 2C).

**Figure 3:**
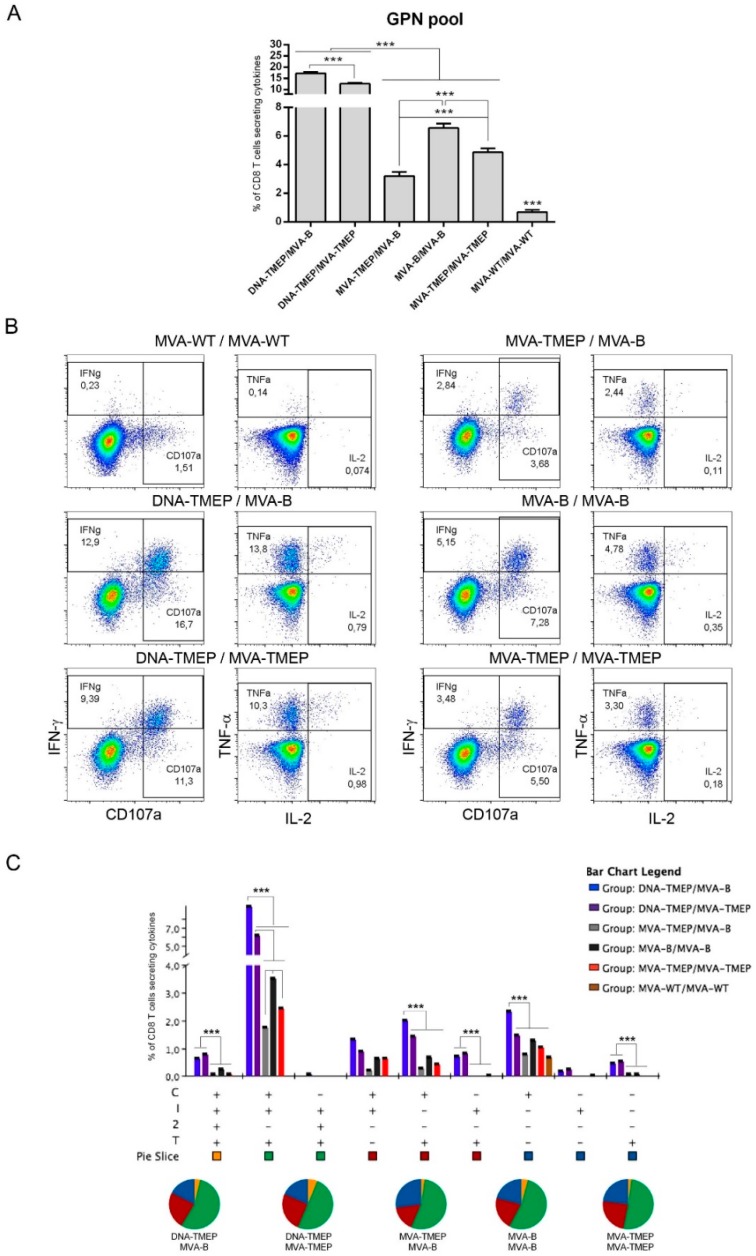
Polyfunctional Gag-Pol-Nef (GPN)-specific CD8 T cell responses induced in spleen after prime/boost immunization of mice with different DNA and MVA vectors expressing the TMEP-B protein. (**A**) Magnitude of the GPN-specific CD8 T cell responses determined at 10 days post-boost by ICS assay after the stimulation of splenocytes from immunized mice with the HIV-1 clade B consensus GPN pool. The total value in each group indicates the sum of the percentages of CD8^+^ T cells secreting IFN-γ and/or IL-2 and/or TNF and/or expressing CD107a against the HIV-1 GPN pool. Data are background-subtracted. (**B**) Flow cytometry profiles of the vaccine-induced CD8 T cell response against the GPN pool in the different immunization groups. (**C**) Polyfunctional profile of the GPN-specific CD8 T cell response. The percentages of the functionally distinct cell populations within the total CD8 T cells are represented on the *y* axis, while positive combinations of the responses are shown on the *x* axis. Responses are grouped and color-coded based on the number of functions. C: CD107a; I: IFN-γ; 2: IL-2; T: TNF. Data from one experiment representative of two performed are shown and 95% CI is represented. ***, *p* < 0.001.

Regarding GPN-specific CD8 T cell responses (Figure 3), animals immunized with the heterologous DNA/MVA combinations again elicited significantly higher GPN-specific CD8 T cells than those induced in animals immunized with the homologous MVA/MVA combinations (*p* < 0.001). Moreover, immunization with the heterologous insert regimen (DNA-TMEP/MVA-B) induced higher GPN-specific CD8 responses than the homologous insert regimen (DNA-TMEP/MVA-TMEP) (*p* < 0.001), indicating in this case a positive effect of the use of the heterologous inserts TMEP-B and GPN on the GPN-specific responses compared to identical TMEP-B antigen both in prime and boost. In the groups immunized with homologous MVA/MVA combinations, the regimen MVA-B/MVA-B elicited the highest GPN-specific CD8 T cell response followed by MVA-TMEP/MVA-TMEP and MVA-TMEP/MVA-B (*p* < 0.001) (Figure 3A). Representative flow cytometry profiles of the GPN-specific CD8 T cell responses induced by the different immunization regimens are shown in Figure 3B. With regard to the functional profile, nine positive GPN-specific CD8 T cell populations were elicited after immunization with the different prime/boost protocols (Figure 3C). In all groups, GPN-specific responses were highly polyfunctional, with more than 50% of responding CD8^+^ T cells exhibiting three or four functions. CD8^+^ T cells producing CD107a + IFN-γ + TNF was the most representative population induced (Figure 3C).

The contribution of Gag and GPN pools to the overall specific CD8 T cell response was different in both DNA/MVA heterologous combinations. In MVA-B-boosted mice the ranking was GPN (17.3%) > Gag (11.8%) whereas in MVA-TMEP-boosted mice was the opposite (Gag (22.7%) > GPN (12.6%)).

Additionally, we also examined the phenotype of the HIV-1-specific CD8 T cell response by measuring the expression of CD127 and CD62L surface markers, which allows the definition of the following T cell memory subpopulations: T central memory (TCM; CD127^+^CD62L^+^), T effector memory (TEM; CD127^+^CD62L^−^) and T effector (TE; CD127^−^CD62L^−^) (Figure S1A). Gag- and GPN-specific CD8 T cell responses were mostly of TEM phenotype in all immunization groups (Figure S1B).

**Figure 4:**
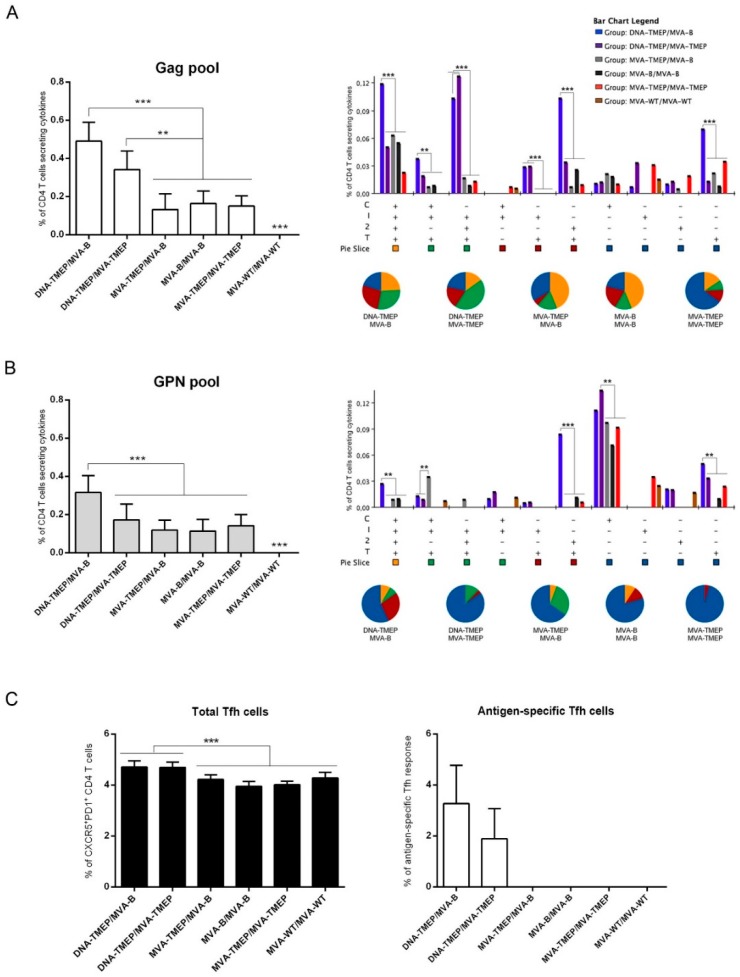
Polyfunctional HIV-1-specific CD4 T cell responses induced in spleen after prime/boost immunization of mice with different DNA and MVA vectors expressing TMEP-B protein. (**A**,**B**) Magnitude (left panels) and polyfunctional profile (right panels) of the Gag-specific (A) or GPN-specific (B) CD4 T cell responses determined at 10 days post-boost by ICS assay after the stimulation of splenocytes from immunized mice with HIV-1 clade B consensus Gag or GPN peptide pools. Left panels: The total value in each group represents the sum of the percentages of CD4^+^ T cells secreting IFN-γ and/or IL-2 and/or TNF and/or expressing CD107a against HIV-1 Gag or GPN peptide pools. Data are background-subtracted. Right panels: Polyfunctional profile of the Gag- or GPN-specific CD4 T cell responses. The percentages of the functionally distinct cell populations within the total CD4 T cells are represented on the *y* axis, while positive combinations of the responses are shown on the *x* axis. Responses are grouped and color-coded based on the number of functions. C: CD107a; I: IFN-γ; 2: IL-2; T: TNF. (**C**) Total (left panel) or Gag-specific (right panel) T follicular helper (Tfh) cells determined at 10 days post-boost by ICS assay in the splenocytes from immunized animals. Left panel: Magnitude of the total CD4 T cells with Tfh phenotype (CXCR5^+^PD1^+^) in non-stimulated splenocytes. Right panel: Magnitude of the Gag-specific Tfh cells. The total value in each group indicates the sum of the percentages of Tfh^+^ cells producing IL-4 and/or IFN-γ and/or IL-21 and/or expressing CD154 (CD40L) against the Gag pool. Data are background-subtracted. Data from one experiment representative of two performed are shown and 95% CI is represented. **, *p* < 0.005; ***, *p* < 0.001.

Regarding CD4 T cell subset (Figure 4A,B), heterologous DNA/MVA regimens elicited higher Gag- and GPN-specific CD4 T cell responses compared with homologous MVA/MVA combinations and specifically mice immunized with the combination DNA-TMEP/MVA-B elicited a higher HIV-1-specific CD4 T cell response compared to the group receiving DNA-TMEP/MVA-TMEP. In both heterologous DNA/MVA groups, the individual contribution of each pool to the total CD4 response was Gag > GPN. No statistical difference was observed in the response induced by the groups immunized with the homologous MVA/MVA combinations (Figure 4A,B, left panels). Flow cytometry plots of Gag- and GPN-specific CD4 T cell responses induced by the different immunization regimens are shown in Figure S2. With respect to the functional profile, a difference was observed between Gag- and GPN-specific responses. While the Gag-specific CD4 T cell response was highly polyfunctional with CD107a + IFN-γ + IL-2 + TNF and IFN-γ + IL-2 + TNF being the most representative populations induced (Figure 4A, right panel), GPN-specific CD4 T cell response was monofunctional with single CD107a^+^ cells being the most representative population induced (Figure 4B, right panel).

#### 3.2.2. HIV-1-Specific Tfh Cell Immune Responses Elicited in Mice

CD4 T follicular helper (Tfh) cells are specialized in providing help to B cells, which are critical for the development and maintenance of germinal center (GC) reactions, an essential process that induces the generation of long-lived high affinity antibodies. Since the development of broadly neutralizing antibodies (bNAbs) has been correlated with the frequency and quality of Tfh cells [22,23], we decided to characterize this specific cellular subset in the spleen of immunized animals. Therefore, splenocytes were non-stimulated (RPMI) or stimulated ex vivo for 6 h with the different HIV-1 clade B consensus peptide pools. Double positive CXCR5^+^PD-1^+^ population gated on CD4^+^ T cells defined total Tfh cells, whereas percentages of Tfh cells that produced IL-4 and/or IL-21 and/or IFN-γ and/or expressed CD154 (CD40L) established the HIV-1-specific Tfh responses (Figure S3A). The percentages of total CD4 Tfh cells were higher in mice that received the heterologous DNA/MVA combinations compared to homologous MVA/MVA regimens (*p* < 0.001) (Figure 4C, left panel). HIV-1-specific Tfh cells were only detected against the Gag pool in the groups of mice immunized with the heterologous DNA/MVA combinations, with no statistical difference between both groups (Figure 4C, right panel). Flow cytometry plots of Gag-specific CD4 Tfh cells induced by the different immunization regimens are shown in Figure S3B.

No FLAG-specific CD4, CD8 or Tfh responses were detected, reinforcing the use of the FLAG tag to allow the detection of TMEP-B protein in cells without triggering a tag-specific immune response when tested in vivo.

In summary, as shown in Figure 2A, Figure 3A and Figure 4A,B, vaccine-elicited T cell immune responses were mainly mediated by the CD8 T cell subset with heterologous DNA/MVA combinations inducing significantly higher magnitudes compared to homologous MVA/MVA regimens (*p* < 0.001).

#### 3.2.3. Effect of DNA Prime on the HIV-1 Env-Specific T Cell Responses in Mice Receiving MVA-B Boost

**Figure 5:**
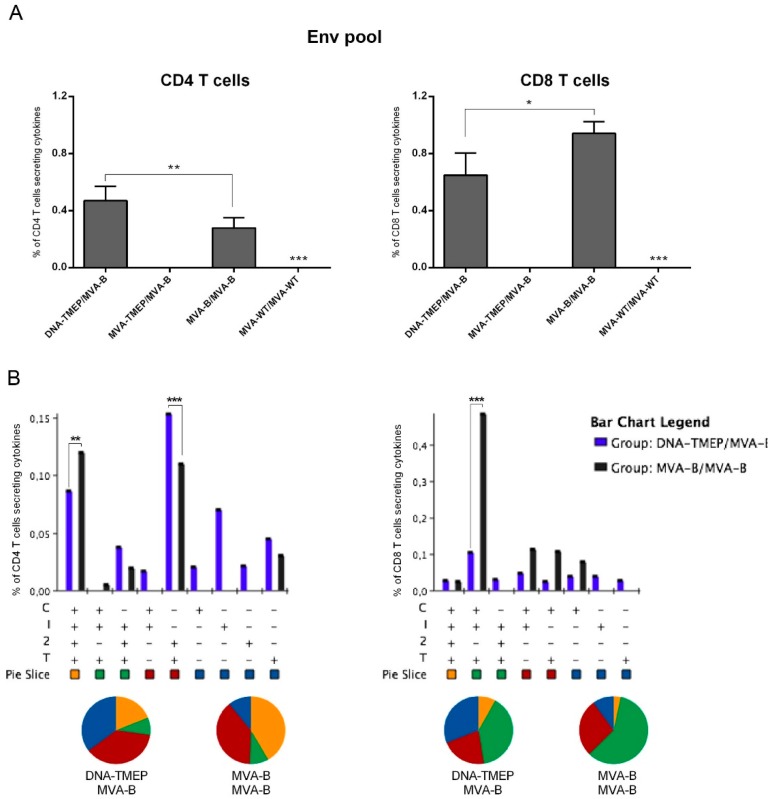
Polyfunctional Env-specific CD4 and CD8 T cell responses induced in spleen after prime/boost immunization of mice with the MVA-B vector. (**A**) Magnitude of the Env-specific CD4 (left panel) or CD8 (right panel) T cell responses determined at 10 days post-boost by ICS assay after the stimulation of the splenocytes from immunized mice with the HIV-1 clade B consensus Env pool. The total value in each group represents the sum of the percentages of CD4^+^ or CD8^+^ T cells secreting IFN-γ and/or IL-2 and/or TNF and/or expressing CD107a against the HIV-1 Env pool. Data are background-subtracted. (**B**) Polyfunctional profile of the Env-specific CD4 (left panel) or CD8 (right panel) T cell responses. The percentages of the functionally distinct cell populations within the total CD4 or CD8 T cells are represented on the *y* axis, while positive combinations of the responses are represented on the *x* axis. Responses are grouped and color-coded based on the number of functions. C: CD107a; I: IFN-γ; 2: IL-2; T: TNF. Data from one experiment representative of two performed are shown and 95% CI is represented. *, *p* < 0.05; **, *p* < 0.005; ***, *p* < 0.001.

We next evaluated the magnitude and quality of the T cell response induced against the Env antigen, only expressed when the MVA-B vector was administered. As shown in Figure 5A, Env-specific T cell responses were detected in mice primed with DNA-TMEP or MVA-B and boosted with MVA-B vector. No Env-specific CD4 or CD8 T cell responses were induced when a homologous vector expressing a heterologous insert was used (MVA-TMEP/MVA-B). In mice immunized with the DNA-TMEP/MVA-B combination, the Env-specific responses were evenly distributed between CD4 and CD8 T cell compartments while in mice immunized with the MVA-B/MVA-B regimen the responses were polarized to the CD8 compartment (Figure 5A). Flow cytometry plots of Env-specific CD4 and CD8 T cells induced by the different immunization regimens are shown in Figure S4A,B. When we evaluated the functional profile of the Env-specific T cell response, nine and eight different positive CD4 (Figure 5B, left panel) or CD8 (Figure 5B, right panel) T cell populations were induced, respectively. Env-specific CD4 T cells were more polyfunctional in animals immunized with MVA-B/MVA-B compared with animals immunized with DNA-TMEP/MVA-B, with 50% (MVA-B/MVA-B) or 30% (DNA-TMEP/MVA-B) of responding CD4^+^ T cells exhibiting three or four functions. CD4 T cells producing CD107a + IFN-γ + IL-2 + TNF or IL-2 + TNF were the most representative populations induced (Figure 5B, left panel). Similarly, Env-specific CD8 T cells were also more polyfunctional in mice immunized with MVA-B/MVA-B compared to mice immunized with the DNA-TMEP/MVA-B regimen, with 60% (MVA-B/MVA-B) or 45% (DNA-TMEP/MVA-B) of responding CD8^+^ T cells exhibiting three or four functions. CD8 T cells producing CD107a + IFN-γ + TNF was the most representative population induced (Figure 5B, right panel). Env-specific CD8 T cell responses were mostly of TEM phenotype in all immunization groups (Figure S4C). No Env-specific Tfh response was detected in any of the immunization groups.

Overall, these immunogenicity results revealed that heterologous DNA/MVA immunization regimens induced higher HIV-1-specific T cell immune responses (CD4, CD8 and Tfh cells) compared with homologous MVA/MVA combinations, with a clear polarization to the CD8 T cell compartment. Furthermore, in DNA-TMEP/MVA-B-immunized animals (heterologous inserts), the CD8^+^ T cell responses were mostly induced against the GPN pool while in the DNA-TMEP/MVA-TMEP group (homologous inserts) the Gag-specific response was two-fold higher than the GPN-specific responses. Env-specific CD8 T cell responses were also detected in animals primed with either DNA-TMEP or MVA-B and boosted with the MVA-B vaccine vector.

#### 3.2.4. Vector MVA-Specific CD8 T Cell Immune Response

**Figure 6:**
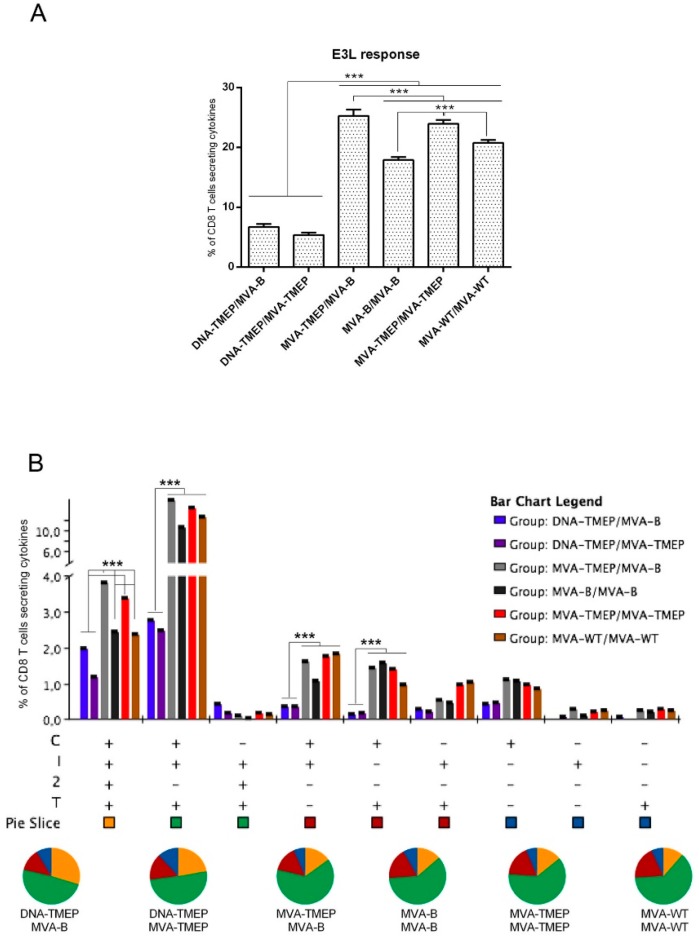
Polyfunctional vaccinia virus (VACV) E3-specific CD8 T cell immune response induced in spleen after prime/boost immunization of mice with different DNA and MVA vectors expressing the TMEP-B protein. (**A**) Magnitude of the VACV E3-specific CD8 T cell immune response determined at 10 days post-boost by ICS assay after the stimulation of splenocytes from immunized mice with VACV E3 peptide. The total value in each group represents the sum of the percentages of CD8^+^ T cells secreting IFN-γ and/or IL-2 and/or TNF and/or expressing CD107a against VACV E3 peptide. Data are background-subtracted. (**B**) Polyfunctional profile of the VACV E3-specific CD8 T cell response. The percentages of the functionally distinct cell populations within the total CD8 T cells are represented on the *y* axis, while different combinations of the responses are shown on the *x* axis. Responses are grouped and color-coded based on the number of functions. C: CD107a; I: IFN-γ; 2: IL-2; T: TNF. Data from one experiment representative of two performed are shown and 95% CI is represented. ***, *p* < 0.001.

Finally, we also evaluated the vector MVA-specific response elicited by the different immunization protocols measuring the response against VACV E3 peptide for the CD8 T cell subset. As shown in Figure 6A, the groups receiving the heterologous DNA/MVA regimens elicited the lowest E3-specific responses (*p* < 0.001), which was consistent with the one single virus immunization dose used in these groups. In homologous MVA/MVA combinations, MVA-TMEP-primed animals induced higher E3-specific CD8 T cell responses compared with MVA-B- or MVA-WT-primed animals (*p* < 0.001) (Figure 6A). In spite of the differences in magnitude, a similar functional profile of anti-vector response was detected in all the immunization groups (Figure 6B). VACV E3-specific CD8 T cells were highly polyfunctional, with more than 70% of responding CD8^+^ T cells exhibiting three or four functions. CD8^+^ T cells simultaneously producing CD107a + IFN-γ + IL-2 + TNF or CD107a + IFN-γ + TNF were the most representative populations induced by the different prime/boost immunization protocols (Figure 6B). The lower anti-MVA vector-specific response induced by DNA/MVA combinations over the other MVA/MVA regimens is an added advantage, as there will be less interference with the further expansion of specific T cell responses in case additional viral booster doses are needed.

## 4. Discussion

It has been reported that vaccine candidates engineered to trigger adaptive immune responses that favorably target conserved antigenic regions of viral susceptibility might enable better immune control after preventive and therapeutic vaccination for HIV-1, and circumvent the immunodominance of variable non-protective epitopes observed when full-length HIV-1 protein vaccines are used [6,24,25]. We have recently designed a multiepitopic T cell protein based on the use of highly conserved beneficial regions within the Gag, Pol and Nef HIV-1 proteome that have been associated with HIV-1 control defined by human immune reactivity data [10]. When combined in prime/boost with the HIV-1 vaccine MVA-B co-expressing the full-length GPN and the monomeric Env gp120, the DNA-TMEP vector induced significantly higher HIV-1-specific T cell responses than the DNA-GPN vector expressing the full-length GPN antigen. These results suggested that the use of optimized TMEP-B immunogen as a prime directed the immune responses preferentially to those T cell clones specific for conserved protective epitopes that were difficult to prime when full-length antigens were administered due to the immunodominance hierarchy. Previous studies have shown that vaccination strategies using heterologous vectors and inserts improved the T cell breadth and coverage. These results were observed using either full-length Env or Gag proteins that shared multiple highly conserved potential T cell epitopes (PTEs) but diverged at non-conserved sequences [26,27] or combining heterologous inserts based on conserved elements (CE) with full-length antigens [5,6,25]. However, it has also been reported that homologous CE-based inserts exhibited stronger Gag-specific responses than heterologous insert combination with full-length antigen [28] or even when delivered by different vectors [4,7].

To extend our previous findings, here we vectored the TMEP-B protein in the attenuated MVA strain (MVA-TMEP) and evaluated whether prime-boost injections with homologous (MVA/MVA) or heterologous (DNA/MVA) vectors expressing homologous (TMEP/TMEP) or heterologous (TMEP/GPN) inserts differentially impact the magnitude, function and antigen recognition pattern of activated T cells. We observed that heterologous vector regimen (DNA/MVA) was superior to the homologous combination (MVA/MVA) in inducing HIV-1-specific T cell-mediated immune responses, independently of the insert expressed. In both regimens, the responses were mainly mediated by the CD8 T cell subset. The preferential induction of HIV-1-specific CD8 over CD4 T cell responses can be explained by the nature of the antigen expressed, since the HIV-1 regions included in TMEP-B construct were designed to be efficiently processed by the proteasome and mainly presented by the major histocompatibility complex class I (MHC I) to CD8 T cells.

In heterologous DNA/MVA vector combinations, the overall magnitude of the HIV-1-specific (Gag + GPN) CD8 T cell responses was higher when mice were immunized with the same insert (35.3% in DNA-TMEP/MVA-TMEP vs. 29.1% in DNA-TMEP/MVA-B), although in both groups potent and polyfunctional Gag- and GPN-specific CD8 T cell responses with an effector memory phenotype were induced. In DNA-TMEP/MVA-B-immunized animals, the CD8^+^ T cell responses were polarized to the GPN pool (17.3% against GPN vs. 11.8% against Gag) whereas in the DNA-TMEP/MVA-TMEP group the Gag-specific responses were two-fold higher than the GPN-specific responses (22.7% against Gag vs. 12.6% against GPN). HIV-1-specific CD4 and Tfh responses, mainly directed against Gag, were also detected in DNA-TMEP/MVA-TMEP- and DNA-TMEP/MVA-B-immunized animals. The significant expansion of Gag-specific CD8 T cell responses as well as the induction of Gag-specific CD4 and Tfh responses using a homologous insert regimen could be beneficial in terms of vaccine efficacy since experimental evidence from several studies has linked Gag-specific T cell responses with better viral control and slower disease progression [29,30,31,32,33,34].

In homologous MVA/MVA vector regimens, the HIV-1-specific CD8 T cell responses were mainly directed against the GPN pool, independently of the insert expressed. Homologous insert regimens (MVA-B/MVA-B and MVA-TMEP/MVA-TMEP) elicited higher GPN-specific CD8 T cell responses than heterologous insert combination (MVA-TMEP/MVA-B), while Gag-specific CD8 T cell responses were similar in all MVA/MVA groups. The HIV-1-specific CD4 T cell responses were more balanced and no differences were detected between groups. Similar results were reported by Kaufman et al. in rhesus monkeys primed and boosted with recombinant adenovirus vectors expressing homologous or heterologous HIV-1 Gag sequences that were optimized to focus responses on highly conserved epitopes. They observed comparable responses directed to specific regions of the Gag protein without evidence of expanded breadth or improved coverage in the heterologous insert group, suggesting that other factors unrelated to the antigen could contribute to the immunodominance profiles of vaccine-induced cellular responses [35].

In addition, we selectively evaluated the Env-specific T cell responses in those groups immunized with MVA-B since this vector not only expresses the related full-length GPN antigen but also the monomeric Env gp120. The overall magnitude of Env-specific T cell responses elicited by the homologous MVA-B/MVA-B regimen (two doses of Env) was similar to that detected in the DNA-TMEP/MVA-B group (one dose of Env), indicating that either Gag- and GPN-specific T cell responses primed by the DNA-TMEP vector or the absence of anti-vector specific responses, might positively impact the Env-specific response. In DNA-TMEP/MVA-B-immunized animals, balanced CD4 and CD8 T cell responses were detected whereas in the MVA-B/MVA-B group a preferential CD8 T cell response was observed. Interestingly, no Env-specific T cell responses were detected in animals receiving the heterologous insert regimen MVA-TMEP/MVA-B (one dose of Env), suggesting that when TMEP-B is vectored by MVA, the potential positive effect of priming Gag- and GPN-specific clones is obscured by the presence of large amounts of viral antigens.

Overall, our results revealed the induction of HIV-1-specific T cell responses by the multiepitopic protein TMEP-B when vectored in both DNA and MVA and supported their use as T cell components in combined vaccination strategies for HIV-1 prevention and/or therapy. Based on our previous phase I clinical trials, both prophylactic and therapeutic with the vaccine MVA-B where moderate T cell immune responses were induced, the results reported here suggest that the combination of TMEP vectors together with MVA-B should help to further expand the HIV-1-specific CD8 T cell immune responses in vaccinees, a process which might be beneficial to control HIV-1 infections, particularly in those individuals under cART-treatment.

## Supplementary Materials

The following are available online at https://www.mdpi.com/2076-393X/7/3/57/s1. Figure S1: Gag- and GPN-specific CD8 T cell memory phenotype induced by the different immunization regimens. (A) Expression of CD127 and CD62L surface markers defines the following T cell memory subpopulations: T central memory (TCM; CD127^+^CD62L^+^), T effector memory (TEM; CD127^+^CD62L^−^) and T effector (TE; CD127^−^CD62L^−^). (B) Flow cytometry plots of vaccine-induced CD8 T cell memory phenotype against the Gag (left column) or GPN (right column) pools in the different immunization groups. Figure S2: Flow cytometry plots of Gag- (A) and GPN- (B) specific CD4 T cell responses. Figure S3: Gag-specific CD4 Tfh cell responses induced by the different immunization regimens. (A) Flow cytometry strategy for the identification of total and HIV-1-specific Tfh cells. CD4^+^CD8^−^ cells were gated on lymphocytes/singlets/live cells and analyzed based on the expression of CXCR5 and PD-1 markers. The double positive CXCR5^+^PD-1^+^ population was used to identify total Tfh cells. HIV-1-specific Tfh cells were determined by the percentage of Tfh cells secreting IFN-γ and/or IL-4 and/or IL-21 and/or expressed CD40L after the HIV-1 peptide pool stimulation in comparison with non-stimulated cells (RPMI). FSC-A: forward-scatter area; SSC-A: side scatter area; FSC-H: forward-scatter height. (B) Flow cytometry plots of Gag-specific CD4 Tfh cells. Figure S4: Env-specific T cell response and memory phenotype induced by the different immunization regimens. (A, B) Flow cytometry plots of Env-specific CD8 (A) or CD4 (B) T cell responses. (C) Flow cytometry plots of vaccine-induced CD8 T cell memory phenotype against the Env pool.
