# Supplementary figures and images for: Induction of Broad and Polyfunctional HIV-1-Specific T Cell Responses by the Multiepitopic Protein TMEP-B Vectored by MVA Virus
Source: Vaccines (Basel). 2019 Jun 29;7(3):57. doi: 10.3390/vaccines7030057 (PMC6789790; doi:10.3390/vaccines7030057)

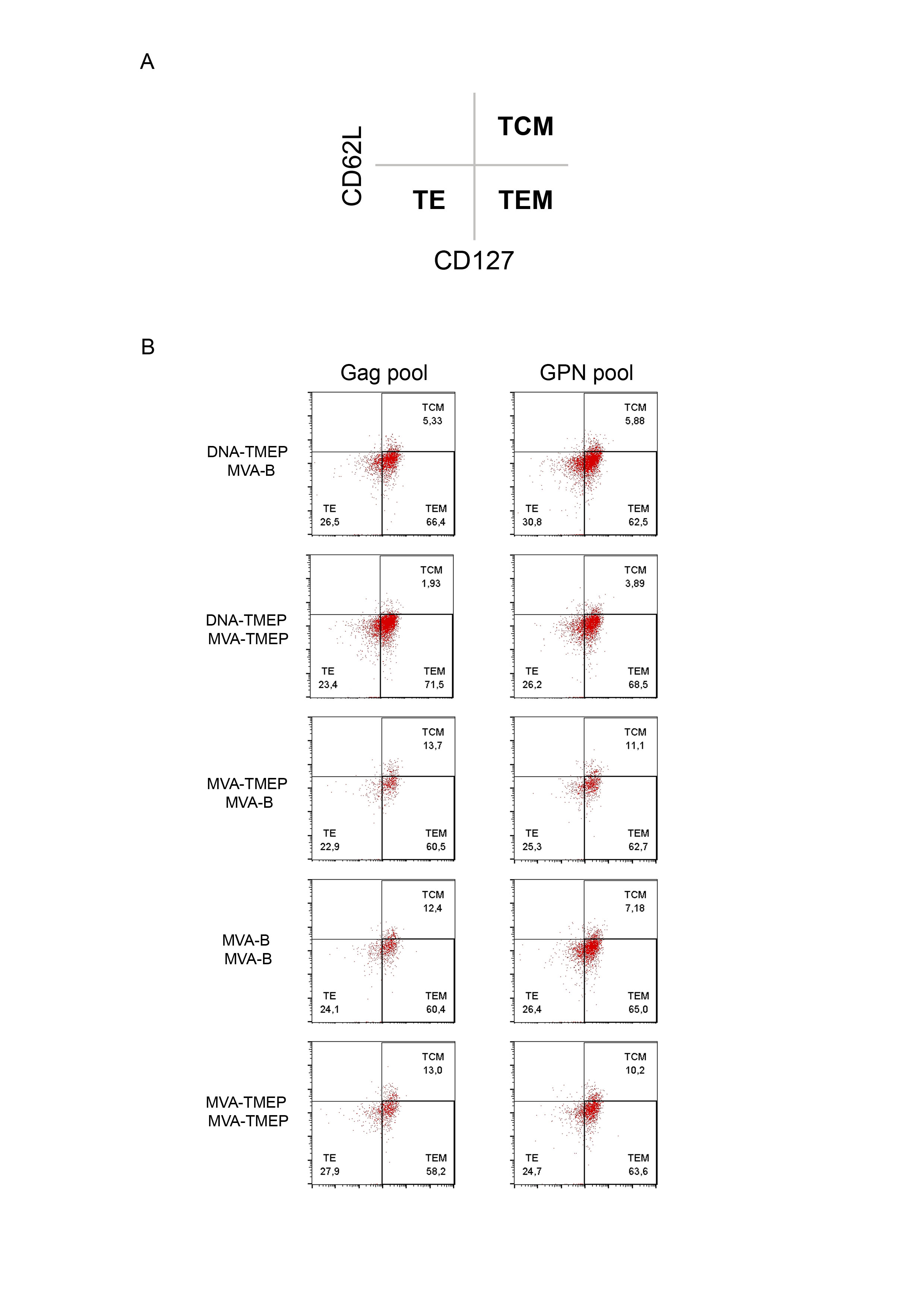

Supplement: Supplementary file 1 [file vaccines-07-00057-s001.zip › figure S1.jpg]

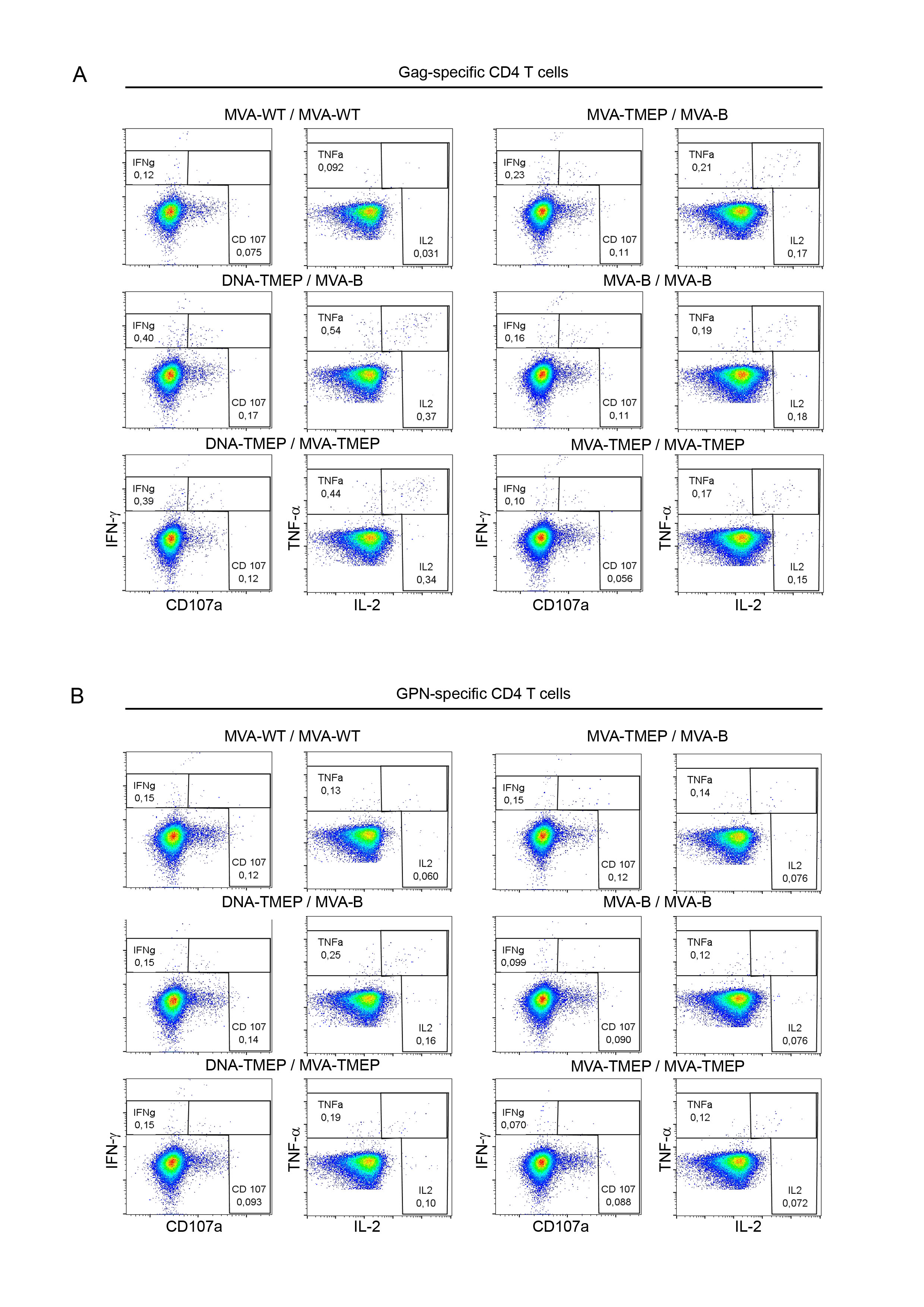

Supplement: Supplementary file 1 [file vaccines-07-00057-s001.zip › figure S2.jpg]

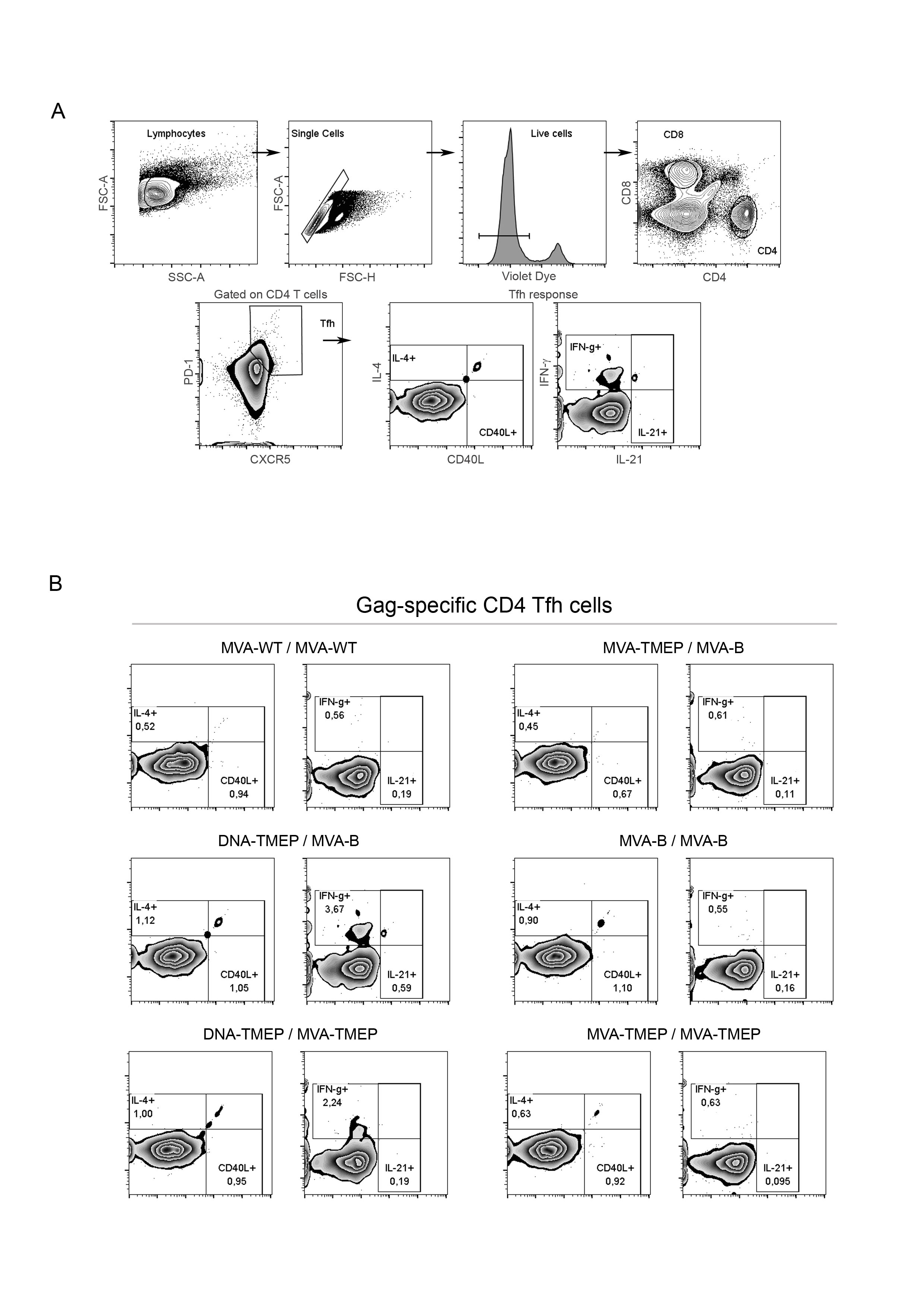

Supplement: Supplementary file 1 [file vaccines-07-00057-s001.zip › figure S3.jpg]

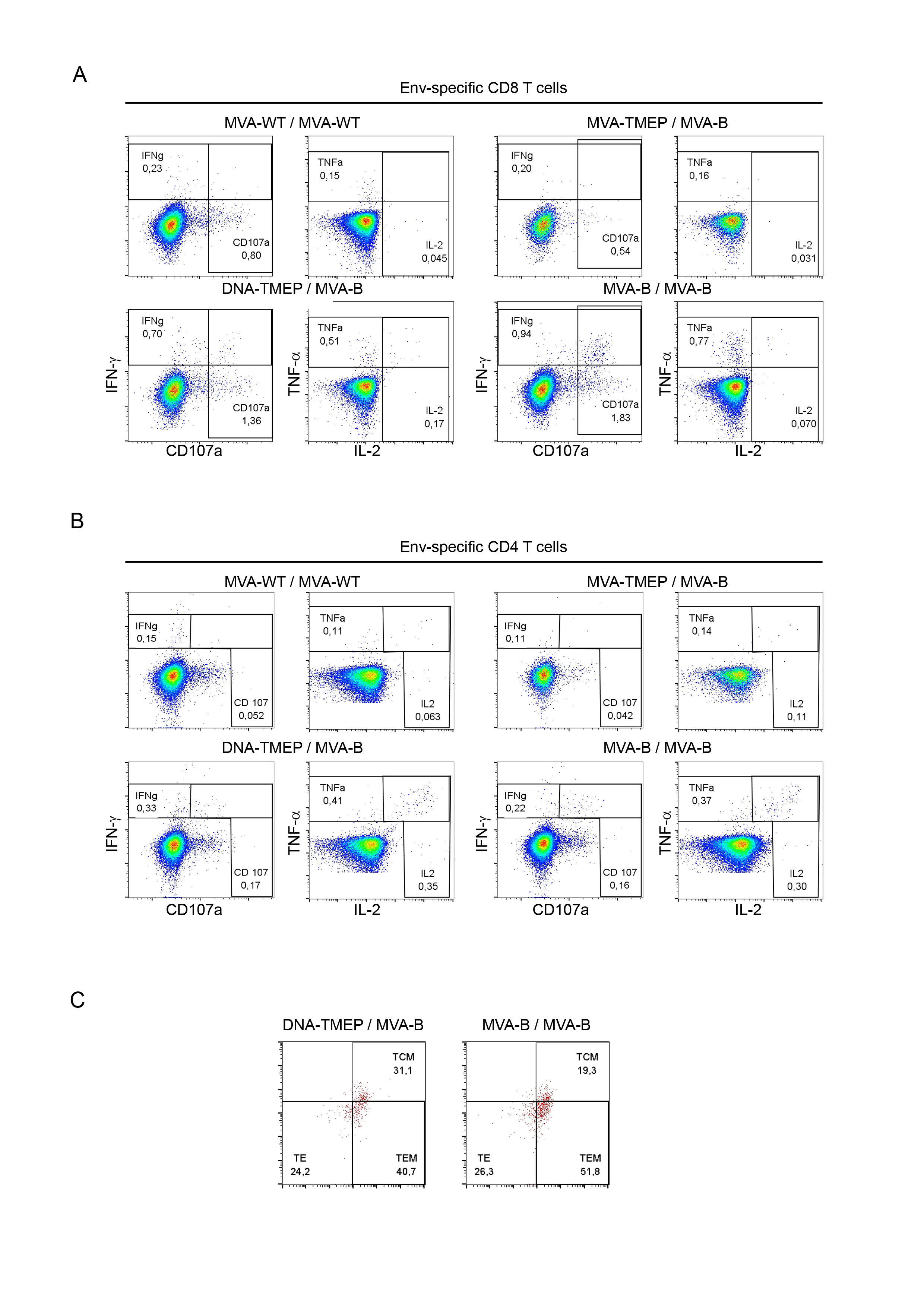

Supplement: Supplementary file 1 [file vaccines-07-00057-s001.zip › figure S4.jpg]
